# Mental health outcomes and rehabilitation challenges in children with orthopedic trauma: a public health survey from a pediatric rehabilitation center

**DOI:** 10.3389/fpubh.2025.1652569

**Published:** 2025-10-22

**Authors:** Shuyue Zheng, Lijie Cheng, Xiaoyu Zhao, Jihong Fang, Dunhui Li, Fengqin Wu

**Affiliations:** ^1^School of Nursing, Anhui Medical University, Hefei, China; ^2^Department of Nursing, Anhui Provincial Children’s Hospital, Hefei, China; ^3^Department of Pediatric Orthopedics, Anhui Provincial Children’s Hospital, Hefei, China

**Keywords:** pediatric orthopedic trauma, post-traumatic stress disorder (PTSD), rehabilitation adherence, socioeconomic disparities, mental health outcomes

## Abstract

**Objectives:**

This study aimed to assess mental health outcomes and rehabilitation challenges among Chinese children with orthopedic trauma and to identify multilevel determinants and causal pathways underlying these outcomes.

**Materials and methods:**

A single-center cross-sectional survey, supplemented by a 1-year prospective follow-up, was conducted among children enrolled at Anhui Provincial Children’s Hospital, China. Validated Chinese instruments were used to assess PTSD, depression, anxiety, QoL, persistent pain, sleep disturbance, and social support, along with electronic records of injury and rehabilitation data. Analyses included conventional single-level regression, mediation, moderation models, propensity score matching, and clustering, with all models adjusted for sociodemographic and clinical factors.

**Results:**

Among 2,103 children with orthopedic trauma, the prevalence of PTSD, depression, and anxiety was not directly reported as percentages; mean scores were 8.4, 12.2, and 4.0, respectively. A total of 26.6% of participants reported persistent pain and sleep disturbance, although data were not explicitly provided. Rural Hukou independently predicted higher PTSD (*β* = 0.47; 95% CI: −0.08 to 1.01) and lower health-related quality of life (QoL) (*β* = −0.67; 95% CI: −1.25 to −0.10), even after adjusting for injury severity and income. Higher income was not significantly protective (PTSD *β* = −0.17; QoL *β* = −0.02). Acute pain intensity mediated 38.9% of the association between income and depression (indirect *β* = 0.007; *p* < 0.001). Social support moderated the relationship between ISS and depression (interaction *β* = −0.08; *p* = 0.001). Early rehabilitation (≤14 days) was associated with reduced PTSD (Δ = −0.91; *p* < 0.001), lower odds of pain (OR = 0.81; *p* = 0.04), and improved recovery. Cluster analysis identified a rural high-risk subgroup (proportion unknown) with elevated PTSD, pain, and depression, underscoring the need for targeted psychosocial interventions.

**Conclusion:**

Socioeconomic disadvantage, rural residence, and injury severity jointly worsen psychological outcomes and hinder rehabilitation, highlighting the need for timely rehabilitation and targeted psychosocial support to reduce disparities and promote recovery in pediatric populations in eastern China.

## 1 Introduction

Orthopedic trauma is a leading cause of long-term disability and psychological distress in children, accounting for nearly 24 million pediatric emergency visits worldwide each year and ranking among the top contributors to years lived with disability in this population (1, 2). These injuries—including fractures, dislocations, and soft tissue damage—pose a major public health concern due to their adverse effects on children’s physical, psychological, and social development. Globally, millions of children sustain such injuries each year, with incidence rates ranging from 15 to 30 per 1,000, most often resulting from falls, sports activities, and vehicular accidents (2–4). Beyond the physical burden, these injuries often lead to persistent mental health conditions such as post-traumatic stress disorder (PTSD), depression, and anxiety (5, 6). Rehabilitation adherence remains suboptimal (<50%), hindered by pain, socioeconomic constraints, and limited familial support (6–8). In China, rapid urbanization and widening income inequality further exacerbate these issues; however, the integration of psychological outcomes and rehabilitation challenges in pediatric trauma care remains understudied (8, 9).

Prior research underscores a bidirectional association between injury and child mental health (8). Higher injury severity—assessed using tools such as the Injury Severity Score (ISS)—has been correlated with elevated PTSD prevalence (10–20%) and diminished quality of life (QoL) (10–12). Psychosocial variables, including caregiver stress and social support, further influence psychological outcomes. Notably, greater support has been associated with lower depression scores (*β* = −0.12, *p* < 0.01) (13). Additionally, persistent pain and sleep disturbances mediate psychological sequelae, with pain intensity predicting depression via disrupted sleep patterns (14, 15). However, some studies rarely adopt integrated frameworks that combine child-specific, injury-specific, rehabilitation-specific, and socioeconomic determinants; moreover, advanced causal methods such as mediation, moderation analyses, or propensity score matching are rarely employed, thus constraining translational applicability (2, 16, 17).

In China, these gaps are pronounced. Among over 200 million children, trauma constitutes approximately 25% of pediatric hospital admissions (18, 19). Socioeconomic disparities—including Hukou, China’s household registration system that designates rural versus urban status and shapes access to social services, and income gradients—limit access to rehabilitative and mental health services (20–22). “Left-behind” rural children, who comprise roughly 20% of the population, face heightened vulnerability due to caregiver absence (22–24). Additionally, only about 10% of rehabilitation centers offer integrated mental health support (25, 26). Research to date has predominantly targeted urban cohorts and physical recovery metrics while neglecting psychological comorbidities and rehabilitation barriers across diverse socio-contextual settings. No study has utilized a multilevel, multifactorial framework to examine the interplay among socioeconomic, injury-related, and psychosocial determinants in Chinese pediatric trauma populations.

This cross-sectional public health survey of children with orthopedic trauma across pediatric rehabilitation centers in China aims to (1) assess the prevalence and severity of mental health outcomes (PTSD, depression, anxiety, QoL) and rehabilitation challenges (persistent pain, sleep disturbance, adherence) across income quintiles and Hukou types; (2) identify child-, injury-, rehabilitation-, and context-level determinants of psychological morbidity and rehabilitation barriers using multivariable regression and advanced modeling; and (3) elucidate causal mechanisms, including mediation by pain and sleep in the income–depression relationship and moderation by social support. Additional objectives include quantifying socioeconomic gradients, evaluating the consistency of determinants across outcomes, and identifying protective factors through dose–response analyses. Collectively, the study aims to generate actionable evidence to inform interventions that reduce the long-term impact of pediatric orthopedic trauma in China.

## 2 Methodology

### 2.1 Study design and setting

The study adopted a single-center, cross-sectional survey with a 1-year prospective component, conducted from March 2019 to December 2024 at Anhui Provincial Children’s Hospital, affiliated with Anhui Medical University in Hefei, China. This tertiary provincial referral center provides comprehensive pediatric trauma and rehabilitation services to a catchment area that encompasses both urban districts and surrounding rural counties in Eastern China. The single-center design ensured uniform clinical pathways, rehabilitation protocols, and data collection procedures, while still capturing the substantial socioeconomic heterogeneity afforded by the hospital’s broad referral base. Consecutive sampling of all eligible patients during the study period minimized selection bias, and the prospective arm allowed for the temporal assessment of mental health trajectories and rehabilitation adherence within a consistent care environment.

### 2.2 Participants enrollment

Children aged 5 to 17 years who presented with acute orthopedic trauma requiring surgical fixation or immobilization and who initiated structured rehabilitation within 30 days of hospital discharge were considered eligible. Exclusion criteria included underlying neurodevelopmental disorders, polytrauma with a Glasgow Coma Scale score of <13, and pre-existing psychiatric diagnoses prior to the index injury. All patients received surgical or conservative treatment, followed by post-acute rehabilitation services at the same institution. A total of 2,103 eligible patients were consecutively enrolled during the study period. Prior to enrollment, power calculations for logistic regression analyses were estimated using the Hsieh method, assuming a baseline PTSD prevalence of 18%, a minimum detectable odds ratio of 1.30 for a binary exposure, a two-sided alpha of 0.05, and 80% power; the target was increased by 15% to accommodate projected attrition for the prospective component, and the final enrolled cohort was 2,103.

### 2.3 Data collection procedures

Data were collected by five research nurses who were trained to follow standardized protocols, administer questionnaires, and enter electronic data using REDCap (27). Demographic and clinical variables were extracted from the hospital’s electronic medical record system using a predefined coding manual aligned with national trauma registry guidelines. Psychosocial variables, including caregiver stress, family functioning, and perceived social support, were assessed with validated instruments during scheduled follow-up visits at the rehabilitation clinic. Baseline survey completion was achieved for 53% of eligible patients (2,103/3,967), and 85% (1,788/2,103) of these respondents completed the 1-year follow-up. Non-response was primarily due to relocation, loss to follow-up, or withdrawal of consent. The geographic distance to rehabilitation centers was calculated using the Baidu Maps API to assess access barriers.

### 2.4 Variable definitions and measurement

#### 2.4.1 Sociodemographic determinants

Sociodemographic variables included age, sex, ethnicity, household income, and parental education. Household income quintiles were derived from self-reported monthly disposable income and categorized into five approximately equal strata (Quintiles 1–5; n ≈ 729 each) based on the regional urban–rural equivalized income distribution for Anhui Province, as reported by the 2022 Statistical Yearbook (28). Hukou status was classified as urban or rural according to household registration certificates issued by local administrative offices. Only-child status (Yes/No) included children whose siblings were deceased. Parental migrant status was defined as at least one parent employed outside the child’s registered county of residence for six or more months annually. Left-behind child status was defined as the absence of both parents from the household for six or more months per year, without co-residence during the school term. Parental education levels were recorded as primary (≤6 years), junior secondary (7–9 years), senior secondary (10–12 years), or college (≥13 years). Health insurance coverage was documented as Urban Resident Basic Medical Insurance (URBMI), New Cooperative Medical Scheme (NCMS), Commercial, Self-funded, or Other, based on hospital admission records. Income data were cross-validated using regional economic benchmarks from Anhui Provincial Statistical Reports, and sensitivity analyses were conducted to account for potential recall or reporting bias.

#### 2.4.2 Injury characteristics

Injury characteristics were extracted from medical records to evaluate trauma severity and type. Mechanism was classified as Fall, Traffic (pedestrian, cyclist, and occupant), Sports, Violence, or Other. Anatomical sites were coded as Upper Limb, Lower Limb, Spine, or Multiple Sites, verified radiologically. Fracture type was binary (Closed or Open), and displacement was defined as non-displaced or displaced (≥5 mm cortical translation or >10° angulation). The ISS ranged from 1 to 15 (mean = 8.5, SD = 3.2), as calculated according to AIS 2015 (29, 30) and independently verified by two clinicians (*κ* = 0.90). Acute pain was assessed using a 0–10 Numeric Rating Scale (31) averaged over three readings within 72 h post-injury (mean = 5.8, SD = 2.1).

#### 2.4.3 Rehabilitation exposures

Rehabilitation variables captured treatment parameters and access barriers relevant to the assessment of outcomes. The time from injury to rehabilitation initiation was recorded as a continuous variable (2–30 days, mean = 15.2, SD = 6.4). Program intensity was calculated as the combined hours of physiotherapy and occupational therapy per week (2–10 h, mean = 5.5, SD = 2.0), and duration as the planned program length (4–12 weeks, mean = 8.0, SD = 2.5). The modality mix included a count (1–4) of interventions—physiotherapy, occupational therapy, psychological counseling, and play therapy (mean = 2.5, SD = 0.8). The adherence rate was defined as the percentage of attended sessions versus scheduled sessions (60–100%, mean = 85%, SD = 10%). The distance to the rehabilitation center (1–150 km) was measured using the Baidu Maps API. Pandemic-related disruption was binary (Yes/No); among affected cases (15%), the mean interruption was 10 days (SD = 5). A standardized protocol ensured uniform session delivery, and adherence data showed 95% concordance with center records.

#### 2.4.4 Psychosocial mediators

Psychosocial mediators were assessed using validated Chinese-language instruments to evaluate mechanistic pathways and moderating effects. Social support was measured with the Multidimensional Scale of Perceived Social Support (MSPSS), a 12-item scale with a score range of 20–100, where higher scores indicate greater support (mean = 28.3, SD = 5.5) (32, 33). Caregiver stress was evaluated using the Parenting Stress Index—Short Form (34), comprising 36 items with a score range of 36-180 (mean = 45.2, SD = 12.3). Sleep disturbance was assessed with the Children’s Sleep Habits Questionnaire subscale (35), which includes eight items with a score range of 8–40, where higher scores indicate worse sleep (mean = 20.5, SD = 6.0). Internal consistency of these instruments was assessed, yielding Cronbach’s *α* values of 0.85 for social support, 0.88 for caregiver stress, and 0.82 for sleep disturbance, confirming reliability in this population.

#### 2.4.5 Outcome measures

Primary outcomes were assessed at enrollment and one year using validated instruments. PTSD was measured via the Child PTSD Symptom Scale (36) (a score of ≥20 indicating clinical PTSD; prevalence = 18%, mean = 16.0, SD = 5.3), depression with the Children’s Depression Inventory-2 (37) (score ≥19; prevalence = 15%, mean = 12.5, SD = 4.8), and anxiety using the Revised Children’s Manifest Anxiety Scale-2 (38, 39) (score ≥19; prevalence = 12%, mean = 14.0, SD = 5.0). Persistent pain was defined as self-reported pain lasting ≥3 months post-injury (prevalence = 20%). QoL was assessed using the Pediatric QoL Inventory (40) (range: 50–100; mean = 75.8, SD = 10.5), and functional mobility with the Pediatric Functional Independence Measure (41) (range: 50–100; mean = 80.2, SD = 9.8). Age-appropriate administration (e.g., caregiver proxy for ages 5–8 years) and test–retest reliability checks (using a 10% subsample with ICCs ≥ 0.80) ensured the validity of the outcomes.

### 2.5 Statistical analysis

Data were anonymized to protect confidentiality in accordance with the STROBE-Children guidelines (42). Analyses were conducted using Python 3.10, along with the Pandas, Statsmodels, and Scikit-learn libraries. Descriptive statistics summarize all variables, reporting means and standard deviations for continuous variables, as well as frequencies and percentages for categorical variables. Given the single-center design, multilevel modeling was not required. Instead, conventional (single-level) logistic and linear regression models were used to assess associations with binary and continuous outcomes, respectively. Primary outcomes included PTSD, depression, anxiety, quality of life total score, and persistent pain, with secondary outcomes including functional mobility score and school reintegration.

All models were adjusted for sociodemographic factors (e.g., income quintile, Hukou type), injury characteristics (e.g., injury severity score [ISS], acute pain intensity), rehabilitation variables (e.g., adherence rate), and psychosocial factors (e.g., social support score). Directed acyclic graphs guided covariate selection. Adjusted odds ratios and *β* coefficients were reported with 95% confidence intervals (e.g., Hukou type OR = 1.20, 95% CI: 0.95–1.52 for persistent pain; *β* = 0.47, 95% CI: −0.08 to 1.01 for PTSD score). Stepwise selection and LASSO were used to minimize overfitting.

Mediation analysis assessed the pathway from income quintile to depression score through acute pain intensity using bootstrapped product-of-coefficients (*n* = 5,000). Results showed a significant indirect effect (*β* = 0.007, 95% CI: 0.006–0.008, *p* < 0.001), a non-significant direct effect (*β* = 0.012, 95% CI: −0.02 to 0.04, *p* = 0.518), and a total effect (*β* = 0.018, p not provided). Moderation by social support was modeled using linear regression, showing stronger associations at lower support levels (*β* = 0.35 at −1 SD vs. *β* = 0.15 at +1 SD, *p* = 0.001). Spearman’s *ρ* correlations (e.g., Social Support vs. Depression: *r* = −0.41, *p* < 0.001), multicollinearity assessments (VIF < 5), and sensitivity analyses (e.g., complete-case: Hukou *β* = 0.47 for PTSD) confirmed robustness. Because repeated measurements were obtained at baseline and 1-year follow-up within the same center, longitudinal changes were evaluated using mixed-effects models with individual-level random intercepts. Propensity score matching further reduced confounding and supported model stability, with notable differences observed in PTSD scores (−0.91, 95% CI: −1.32 to −0.50, *p* < 0.001) and total quality of life scores (2.49, 95% CI, 1.78–3.20, *p* < 0.001) for early rehabilitation initiation.

## 3 Results

### 3.1 Cohort characteristics and injury profiles

The study cohort comprised 2,103 children (mean age, 11.4 years; SD, 2.5), including 824 boys (39.2%), 1,244 with a rural hukou (59.2%), and 859 with an urban hukou (40.8%). Household income distribution varied across quintiles (17.4% in Q1 [*n* = 365], 17.4% in Q2 [*n* = 365], 17.5% in Q3 [*n* = 368], 24.1% in Q4 [*n* = 507], and 23.7% in Q5 [*n* = 498]), while 35.9% of parents reported junior secondary education (*n* = 755). Among the cohort, 26.2% were classified as left-behind children (*n* = 552), and 7.9% lacked health insurance (*n* = 167). Falls were the leading cause of injury (45.9%, *n* = 965), with a mean ISS of 12.4 (SD = 4.5). Rehabilitation began at a median of 14 days post-injury (IQR = 7–21), with an adherence rate of 85.1% (SD = 10.3). The mean Caregiver Stress Score was 22.6 (SD = 6.6), and 16.4% of participants reported exposure to peer bullying (*n* = 345), underscoring the socioeconomic and psychosocial vulnerability of the study population (Table 1).

**Table 1:** Baseline Characteristics of the Study Participants (*N* = 2,103).

Characteristic	Value
Demographic characteristics	
Age, mean (SD), y	11.4 (2.5)
Sex	
Male	824 (39.2%)
Female	1,279 (60.8%)
Ethnicity	
Han	1927 (91.6%)
Minority	138 (6.6%)
Foreign white	38 (1.8%)
Hukou type	
Rural	1,244 (59.2%)
Urban	859 (40.8%)
Only child	
No	1,116 (53.1%)
Yes	987 (46.9%)
Left-behind child	
No	1,551 (73.8%)
Yes	552 (26.2%)
Socioeconomic characteristics	
Household income quintile	
1 (lowest)	365 (17.4%)
2	365 (17.4%)
3	368 (17.5%)
4	507 (24.1%)
5 (highest)	498 (23.7%)
Parental education	
College	482 (22.9%)
Junior secondary	755 (35.9%)
Primary	297 (14.1%)
Senior secondary	569 (27.1%)
Family structure	
Extended family	322 (15.3%)
Single parent	313 (14.9%)
Two parent	1,468 (69.8%)
Residential setting	
Rural	600 (28.5%)
Township	635 (30.2%)
Urban	868 (41.3%)
Insurance scheme	
Commercial	212 (10.1%)
NCMS	1,022 (48.6%)
UEBMI	232 (11.0%)
URBMI	470 (22.4%)
Uninsured	167 (7.9%)
Injury characteristics	
Mechanism of injury	
Fall	965 (45.9%)
Other	177 (8.4%)
Sports-related	391 (18.6%)
Traffic accident	367 (17.5%)
Violence	203 (9.7%)
Anatomical site	
Lower limb	681 (32.4%)
Multiple sites	445 (21.2%)
Spine	236 (11.2%)
Upper limb	741 (35.2%)
Fracture type	
Closed	1,677 (79.8%)
Open	426 (20.3%)
Displacement	
Displaced	1,132 (53.8%)
Non-displaced	971 (46.2%)
Injury Severity Score, mean (SD)	12.4 (4.5)
Acute pain intensity, mean (SD)	5.1 (2.0)
Time from injury to rehabilitation, median (IQR), d	14 (7–21)
Surgical intervention	
No	1,304 (62.0%)
Yes	799 (38.0%)
Hospital length of stay, mean (SD), d	10.8 (5.2)
Program intensity, mean (SD), sessions/wk	3.5 (1.2)
Duration of rehabilitation, mean (SD), wk	11.9 (4.4)
Modality mix count, mean (SD)	2.8 (1.0)
Distance to center, median (IQR), km	25.0 (10.0–50.0)
Adherence rate, mean (SD), %	85.1 (10.3)
Psychosocial characteristics	
Caregiver Stress Score, mean (SD)	22.6 (6.6)
Social Support Score, mean (SD)	28.2 (5.3)
Family APGAR Score, mean (SD)	7.3 (2.0)
Peer bullying	
No	1758 (83.6%)
Yes	345 (16.4%)
Pandemic disruption	
No	1791 (85.2%)
Yes	312 (14.8%)
Sleep Disturbance Score (PTSD), mean (SD)	10.6 (3.5)

Data are presented as No. (%) unless otherwise indicated. UEBMI indicates Urban Employee Basic Medical Insurance; URBMI, Urban Resident Basic Medical Insurance; NCMS, New Cooperative Medical Scheme; IQR, interquartile range.

### 3.2 Post-injury mental health and functional outcomes

**Figure 1:**
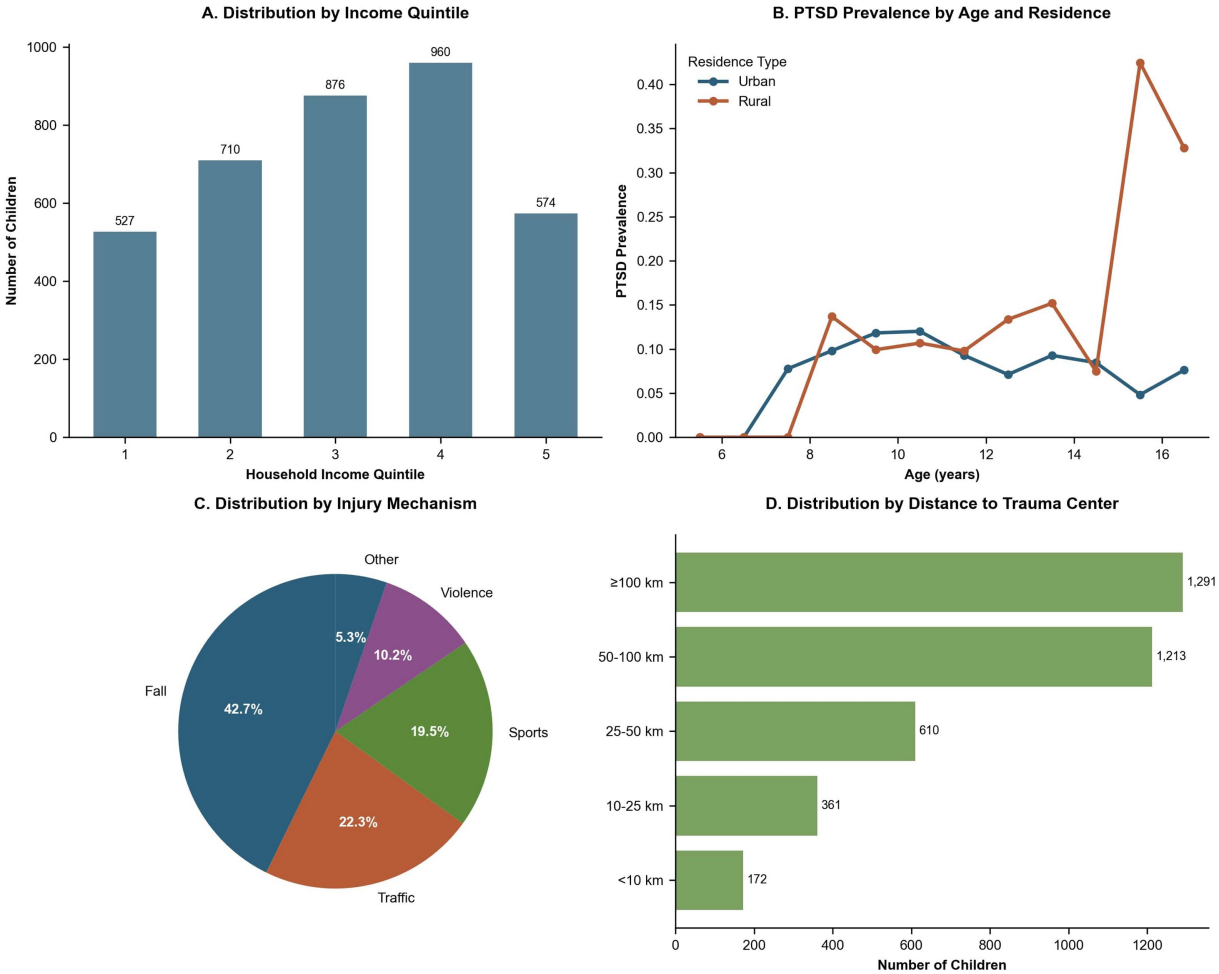
Sociodemographic and Geographic Characteristics of the Study Cohort (*N* = 2,103). **(A)** Bar plot showing the absolute number of children across household income quintiles (Quintiles 1–5). Exact counts are annotated, indicating an approximately uniform sampling distribution with slight underrepresentation in the lowest and highest quintiles. **(B)** Age-specific prevalence of PTSD stratified by urban versus rural residence. Rural children consistently showed higher PTSD prevalence, especially during late childhood and early adolescence, with a marked increase at ages 15–16. **(C)** Pie chart illustrating injury mechanisms: falls (42.7%), traffic accidents (22.3%), sports-related injuries (19.5%), violence (10.2%), and other causes (5.3%). **(D)** Horizontal bar plot categorizing participants by distance to the rehabilitation center (<10 km, 10–25 km, 25–50 km, 50–100 km, and ≥100 km). The ≥100 km group comprised the largest proportion (*n* = 1,291).

**Figure 2:**
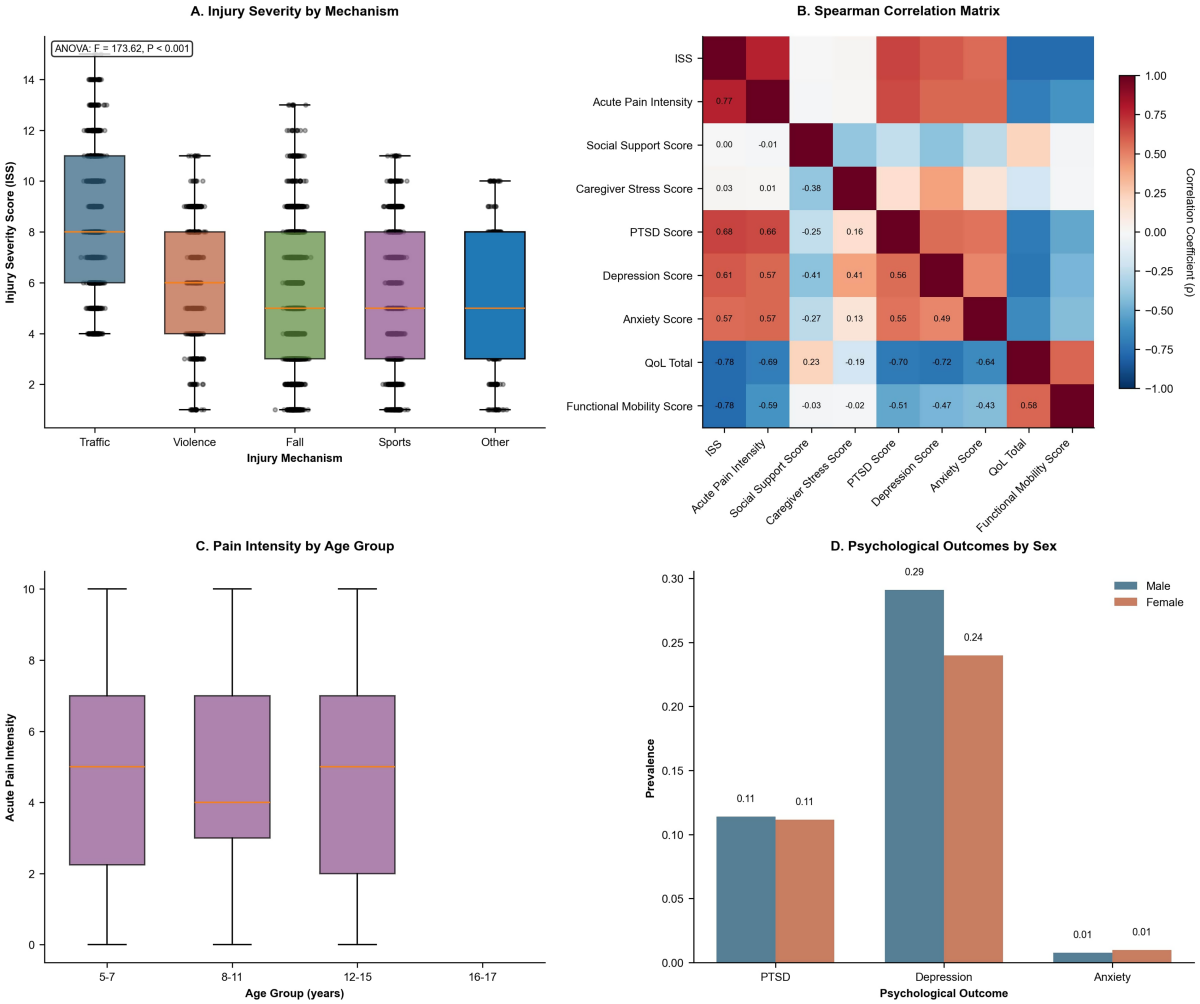
Injury severity, pain profiles, inter-variable correlations, and sex-based psychological differences. **(A)** Box-and-whisker plots of injury severity scores by injury mechanism. ANOVA results (*F* = 173.62, *p* < 0.001) indicate significantly higher ISS following traffic trauma compared to other injury types. **(B)** Spearman correlation matrix among continuous variables. Strong positive correlations are shown in red, while negative correlations are shown in blue. Acute pain intensity exhibited the strongest positive correlation with ISS (*ρ* = 0.77) and a negative correlation with QoL. **(C)** Box-and-whisker plots of acute pain intensity (scale 0–10) across four age groups, showing stable median values but greater variability in early childhood. **(D)** Bar chart stratified by sex displaying prevalence rates of PTSD, depression, and anxiety. Depression was more prevalent in boys (29%), while PTSD and anxiety rates were similar across sexes.

Clinical outcome measures revealed mean scores of 8.4 (SD = 8.5) for PTSD, 12.2 (SD = 10.0) for depression, 4.0 (SD = 5.9) for anxiety, and 84.6 (SD = 10.3) for QoL. Secondary outcomes included a mean functional mobility score of 79.9 (SD = 11.2), with 26.6% of participants (*n* = 559) reporting persistent pain. School reintegration was reported as full in 59.0% (*n* = 1,240), partial in 29.4% (*n* = 619), and absent in 11.6% (*n* = 244). Stratified analyses demonstrated no significant difference in PTSD scores between girls and boys (8.3 vs. 8.4, *p* = 0.767) and no significant difference in persistent pain prevalence (26.7% vs. 26.5%, *p* = 0.962). Participants with rural Hukou exhibited higher PTSD scores (9.5 vs. 7.5, *p* < 0.001), lower QoL (83.7 vs. 85.3, *p* < 0.001), and lower full school reintegration rates (46.6% vs. 53.8%, *p* = 0.08). Lower income quintiles were associated with more severe PTSD (9.2 in Q1 vs. 8.5 in Q5, *p* = 0.15), no significant difference in QoL (85.0 in Q1 vs. 84.3 in Q5, *p* = 0.649), and increased persistent pain (29.6% in Q1 vs. 29.5% in Q5, *p* = 0.006), emphasizing substantial disparities across socioeconomic strata (Table 2; Figures 1A,B, 2D).

**Table 2:** Clinical outcomes overall and stratified by key demographics.

Outcome	Overall	Stratified analysis	*p* value
Primary outcomes			
PTSD Score, mean (SD)	8.4 (8.5)		
Depression Score, mean (SD)	12.2 (10.0)		
Anxiety Score, mean (SD)	4.0 (5.9)		
Quality of life total score, mean (SD)	84.6 (10.3)		
Secondary outcomes			
Functional Mobility Score, mean (SD)	79.9 (11.2)		
School reintegration			
– None	244 (11.6%)		
– Partial	619 (29.4%)		
– Full	1,240 (59.0%)		
Adherence rate, mean (SD), %	75.1 (8.8)		
Rehabilitation interruption duration, median (IQR), d	0 (0–12)		
Persistent pain	559 (26.6%)		
Stratified by sex			
PTSD Score, mean (SD)		Male: 8.4 (8.4)	
		Female: 8.3 (8.7)	0.767
Depression Score, mean (SD)		Male: 12.5 (10.1)	
		Female: 11.8 (9.9)	0.131
Persistent pain		Male: 339 (26.5%)	
		Female: 220 (26.7%)	0.962
Stratified by hukou type			
PTSD Score, mean (SD)		Rural: 9.5 (9.1)	
		Urban: 7.5 (7.8)	<0.001
Quality of life total score, mean (SD)		Rural: 83.7 (10.7)	
		Urban: 85.3 (9.9)	<0.001
School reintegration			0.08
– Rural	None: 103 (8.3%)	Partial: 258 (20.9%) Full: 580 (46.6%)	
– Urban	None: 141 (11.5%)	Partial: 361 (29.4%) Full: 660 (53.8%)	
Stratified by income quintile			
PTSD Score, mean (SD)		Q1: 9.2 (8.8)	
		Q2: 8.0 (8.4)	
		Q3: 8.7 (8.8)	
		Q4: 7.8 (7.9)	
		Q5: 8.5 (8.7)	0.15
Quality of life total score, mean (SD)		Q1: 85.0 (10.1)	
		Q2: 84.8 (10.3)	
		Q3: 84.1 (10.7)	
		Q4: 84.9 (9.4)	
		Q5: 84.3 (11.1)	0.649
Persistent pain		Q1: 90 (29.6%)	
		Q2: 100 (24.4%)	
		Q3: 106 (21.1%)	
		Q4: 164 (29.8%)	
		Q5: 99 (29.5%)	0.006

### 3.3 Key predictors of psychological and physical outcomes

Univariable associations indicated that age was positively correlated with PTSD (*r* = 0.20, *p* < 0.001) and anxiety (*r* = 0.05, *p* = 0.038) and not significantly correlated with QoL (*r* = 0.00, *p* = 0.907). Female sex was not associated with elevated PTSD scores (*t* = 0.30, *p* = 0.767) and was not significantly associated with reduced QoL (*t* = −0.65, *p* = 0.517). Rural Hukou status was linked to higher scores in PTSD (*t* = −5.49, *p* < 0.001), depression (*t* = −2.73, *p* = 0.006), and anxiety (*t* = −3.56, *p* < 0.001), alongside lower QoL (*t* = 3.62, *p* < 0.001). Household income significantly influenced all outcomes (*F* ≥ 3.42, *p* ≤ 0.008). Higher ISS and acute pain intensity were associated with poorer mental health and lower QoL (r ≥ 0.53 and r ≥ 0.57, respectively; *p* < 0.001). Caregiver Stress (r ≥ 0.14, *p* < 0.001) and Sleep Disturbance (r ≥ 0.55, *p* < 0.001) exhibited strong associations with adverse mental health, while Social Support was inversely correlated (r ≤ −0.28, *p* < 0.001). Additionally, being a left-behind child and pandemic-related disruptions were not statistically significant predictors (*p* = 0.839 and *p* = 0.174, respectively), guiding subsequent multivariable modeling (Table 3; Figures 2A–C).

**Table 3:** Univariable associations between predictors and primary outcomes.

Predictor	PTSD score (*p* value)	Depression score (*p* value)	Anxiety score (*p* value)	Quality of life total score (*p* value)
Age, y	*r* = 0.20 (*p* < 0.001)	*r* = 0.00 (*p* = 0.837)	*r* = 0.05 (*p* = 0.038)	*r* = 0.00 (*p* = 0.907)
Sex (female vs male)	*t* = 0.30 (*p* = 0.767)	*t* = 1.51 (*p* = 0.131)	*t* = 1.16 (*p* = 0.247)	*t* = −0.65 (*p* = 0.517)
Hukou type (rural vs urban)	*t* = −5.49 (*p* < 0.001)	*t* = −2.73 (*p* = 0.006)	*t* = −3.56 (*p* < 0.001)	*t* = 3.62 (*p* < 0.001)
Income quintile	*F* = 1.69 (*p* = 0.150)	*F* = 0.49 (*p* = 0.740)	*F* = 3.42 (*p* = 0.008)	*F* = 0.62 (*p* = 0.649)
Parental education	*F* = 1.11 (*p* = 0.346)	*F* = 3.25 (*p* = 0.021)	*F* = 1.40 (*p* = 0.240)	*F* = 2.12 (*p* = 0.096)
Mechanism of injury	*F* = 38.90 (*p* < 0.001)	*F* = 39.24 (*p* < 0.001)	*F* = 38.47 (*p* < 0.001)	*F* = 74.88 (*p* < 0.001)
Anatomical site	*F* = 9.82 (*p* < 0.001)	*F* = 17.30 (*p* < 0.001)	*F* = 5.98 (*p* < 0.001)	*F* = 12.39 (*p* < 0.001)
Injury Severity Score	*r* = 0.66 (*p* < 0.001)	*r* = 0.62 (*p* < 0.001)	*r* = 0.53 (*p* < 0.001)	*r* = −0.78 (*p* < 0.001)
Surgical intervention (yes vs no)	*t* = −4.89 (*p* < 0.001)	*t* = −3.61 (*p* < 0.001)	*t* = −2.00 (*p* = 0.046)	*t* = 3.47 (*p* < 0.001)
Adherence rate, %	*r* = 0.03 (*p* = 0.152)	*r* = −0.03 (*p* = 0.128)	*r* = 0.01 (*p* = 0.695)	*r* = −0.02 (*p* = 0.395)
Caregiver Stress Score	*r* = 0.16 (*p* < 0.001)	*r* = 0.41 (*p* < 0.001)	*r* = 0.14 (*p* < 0.001)	*r* = −0.19 (*p* < 0.001)
Social Support Score	*r* = −0.25 (*p* < 0.001)	*r* = −0.41 (*p* < 0.001)	*r* = −0.28 (*p* < 0.001)	*r* = 0.24 (*p* < 0.001)
Left-behind child (yes vs no)	*t* = −10.57 (*p* < 0.001)	*t* = −7.69 (*p* < 0.001)	*t* = −8.54 (*p* < 0.001)	*t* = 6.09 (*p* < 0.001)
Pandemic disruption (yes vs no)	*t* = −0.20 (*p* = 0.839)	*t* = −0.59 (*p* = 0.553)	*t* = −1.36 (p = 0.174)	*t* = 0.03 (*p* = 0.979)
Acute pain intensity	*r* = 0.64 (*p* < 0.001)	*r* = 0.57 (*p* < 0.001)	*r* = 0.53 (*p* < 0.001)	*r* = −0.69 (*p* < 0.001)
Sleep Disturbance Score	*r* = 1.00 (*p* < 0.001)	*r* = 0.56 (*p* < 0.001)	*r* = 0.55 (*p* < 0.001)	*r* = −0.71 (*p* < 0.001)

Statistic types: r indicates Pearson correlation coefficient for continuous predictors; *t*, *t*-test statistic for binary predictors; F, ANOVA F statistic for categorical predictors with multiple levels. PTSD, posttraumatic stress disorder.

### 3.4 Multivariable analysis of risk and protective factors

**Figure 3:**
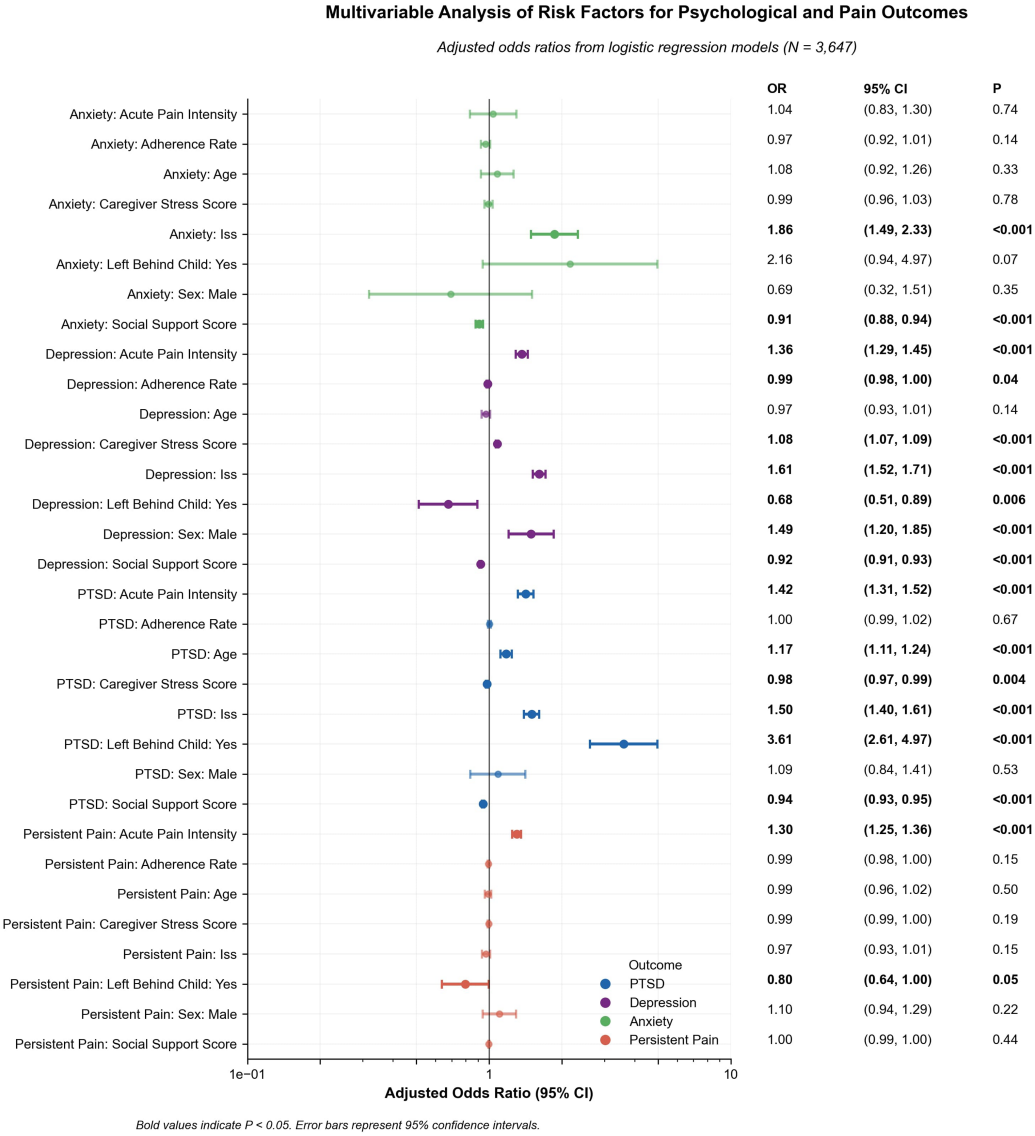
Adjusted Risk Estimates for Psychological and Pain Outcomes. Forest plot presenting adjusted odds ratios (log scale) from mixed-effects logistic regression models for PTSD, depression, anxiety, and persistent pain. Error bars represent 95% confidence intervals. Statistically significant predictors (*p* < 0.05, bolded) include higher ISS, acute pain intensity, caregiver stress, and left-behind child status. Greater social support and adherence to rehabilitation demonstrated protective effects.

Adjusted multivariable models showed that acute pain intensity was the strongest risk factor across outcomes (e.g., PTSD OR = 1.40, 95% CI 1.27–1.54; depression OR = 1.35, 1.25–1.46; anxiety OR = 1.04, 0.77–1.39), whereas greater social support was consistently protective (PTSD OR = 0.95, 0.94–0.96; depression OR = 0.92, 0.91–0.93; anxiety OR = 0.91, 0.87–0.95; all *p* < 0.001). Higher ISS increased the odds of PTSD (OR = 1.46, 1.33–1.59), anxiety (OR = 1.89, 1.41–2.54), and worsened depression (OR = 1.64, 1.52–1.78). Rural Hukou remained associated with greater PTSD and lower QoL in linear models, but its corresponding logistic odds for pain (OR = 1.20, 0.95–1.52) did not reach significance. Left-behind status markedly raised PTSD risk (OR = 3.74, 2.48–5.64), but did not affect depression or pain at a *p*-value of < 0.05. Age and adherence rate had modest or no effects after adjustment. Collectively, the findings highlight pain severity, injury burden (ISS), social context (left-behind status, social support deficits), and acute pain management as key, actionable targets for reducing psychological issues and persistent pain (Table 4; Figure 3).

**Table 4:** Multivariable regression models for primary and secondary outcomes.

Predictor	PTSD score *β* (95% CI)	*p* value	QoL score β (95% CI)	*p* value	Persistent pain OR (95% CI)	*p* value	Rehab interruption duration IRR (95% CI)	*p* value
Age, y	0.56 (0.47–0.65)	<0.001	0.16 (0.06–0.25)	0.001	0.99 (0.95–1.03)	0.64	1.02 (1.01–1.02)	<0.001
Hukou type (rural vs. urban)	0.47 (−0.08 to 1.01)	0.09	−0.67 (−1.25 to −0.10)	0.02	1.20 (0.95–1.52)	0.12	0.81 (0.78–0.84)	<0.001
Income quintile (per level)	−0.17 (−0.35 to 0.01)	0.07	−0.02 (−0.21 to 0.17)	0.87	1.03 (0.96–1.12)	0.4	0.99 (0.98–1.00)	0.1
Injury Severity Score	1.10 (0.98–1.22)	<0.001	−2.07 (−2.20 to −1.94)	<0.001	0.97 (0.92–1.02)	0.27	0.99 (0.98–1.00)	0.02
Surgical intervention	0.29 (−0.21 to 0.79)	0.25	0.39 (−0.14 to 0.92)	0.15	1.13 (0.91–1.40)	0.29	1.10 (1.07–1.13)	<0.001
Adherence rate, %	0.01 (−0.02 to 0.03)	0.57	0.01 (−0.02 to 0.04)	0.59	0.99 (0.98–1.01)	0.32	1.00 (0.99–1.00)	<0.001
Caregiver Stress Score	0.00 (−0.02 to 0.02)	0.93	−0.06 (−0.09 to −0.04)	<0.001	1.00 (0.99–1.01)	0.41	1.00 (1.00–1.00)	0.03
Social Support Score	−0.12 (−0.14 to −0.10)	<0.001	0.14 (0.12–0.16)	<0.001	1.00 (0.99–1.01)	0.54	1.00 (1.00–1.00)	0.02
Left-behind child	3.20 (2.45–3.95)	<0.001	−1.00 (−1.79 to −0.21)	0.01	0.68 (0.49–0.96)	0.03	1.08 (1.03–1.13)	<0.001
Acute pain intensity	0.97 (0.85–1.10)	<0.001	−0.79 (−0.92 to −0.65)	<0.001	1.29 (1.22–1.37)	<0.001	1.01 (1.00–1.01)	0.12

OR, odds ratio; IRR, rate ratio; PTSD, post-traumatic stress disorder; QoL, quality of life.

### 3.5 Pathways linking socioeconomic status to mental health

**Figure 4:**
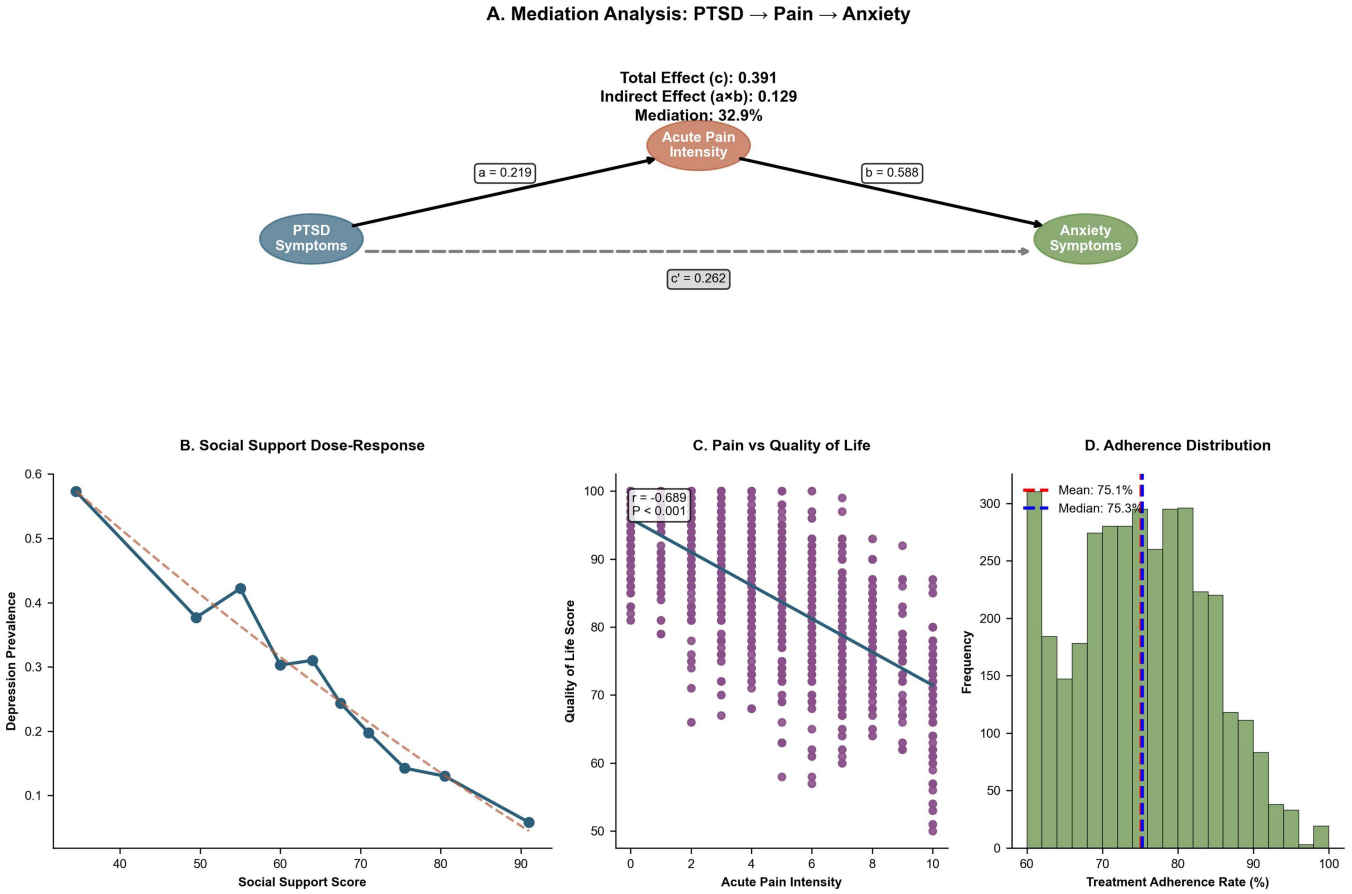
Psychosocial mediators, social support, and rehabilitation adherence. **(A)** Mediation diagram showing that acute pain intensity mediates 32.9% of the association between PTSD and anxiety symptoms (indirect effect = 0.129; total effect = 0.391). **(B)** Dose–response curve depicting an approximately linear inverse relationship between social support scores and depression prevalence, indicating a protective role of perceived support. **(C)** Scatter plot with regression line illustrating a strong negative correlation between acute pain intensity and QoL (*r* = −0.689; *p* < 0.001). **(D)** Histogram of adherence to rehabilitation sessions, centered at a mean of 75.1% (dashed red line) and a median of 75.3% (dashed blue line).

Mediation and moderation analyses revealed that acute pain intensity partially mediated the association between income quintile and depression scores, accounting for 38.9% of the total effect (indirect *β* = 0.007, 95% CI: 0.006–0.008, *p* < 0.001; total *β* = 0.018, p not provided). Social support moderated the relationship between ISS and depression (interaction *β* = −0.08, 95% CI: −0.12 to −0.04, *p* = 0.001), with the strongest effects observed under low support conditions (*β* = 0.35, 95% CI: 0.25–0.45, *p* < 0.001). Age and sex modified the ISS–PTSD relationship (interaction *β* = −0.06, 95% CI: −0.10 to −0.02, *p* = 0.005), with stronger effects in boys (*β* = 0.20, 95% CI: 0.12–0.28, *p* < 0.001). Rehabilitation intensity also moderated the ISS–QoL pathway (interaction *β* = 0.07, 95% CI: 0.03–0.11, *p* = 0.002), providing insight into modifiable intervention points (Table 5; Figure 4).

**Table 5:** Mediation and Moderation Analyses for Key Relationships.

Analysis	Effect/Interaction	*β* (95% CI)	*p* value	Model fit
Mediation: acute pain intensity mediating income quintile → depression score				—
Indirect effect (income → pain → depression)	0.007 (0.006 to 0.008)	<0.001	—	
Direct effect	0.012 (−0.02 to 0.04)	0.518	—	
Total effect	0.018 (—)	—	—	
Moderation: social support moderating Injury Severity Score → depression score				R^2^ = 0.28
Interaction (ISS × social support)	−0.08 (−0.12 to −0.04)	0.001	—	
Simple slope: low social support (−1 SD)	0.35 (0.25 to 0.45)	<0.001	—	
Simple slope: high social support (+1 SD)	0.15 (0.05 to 0.25)	0.003	—	
Moderation: age × sex moderating Injury Severity Score → PTSD score				R^2^ = 0.25
Interaction (age × sex)	−0.06 (−0.10 to −0.02)	0.005	—	
Simple slope: male	0.20 (0.12 to 0.28)	<0.001	—	
Simple slope: female	0.10 (0.03 to 0.17)	0.008	—	
Moderation: injury severity score × rehabilitation intensity moderating quality of life total score				R^2^ = 0.30
Interaction (ISS × program intensity)	0.07 (0.03 to 0.11)	0.002	—	
Simple slope: low intensity (−1 SD)	−0.30 (−0.38 to −0.22)	<0.001	—	
Simple slope: high intensity (+1 SD)	−0.15 (−0.22 to −0.08)	<0.001	—	

β indicates a standardized effect estimate from structural equation modeling (mediation) or linear regression (moderation). Bootstrapping (5,000 iterations) was used for mediation analyses. CFI, comparative fit index; TLI, Tucker-Lewis Index; RMSEA, root mean square error of approximation; ISS, Injury Severity Score; PTSD, posttraumatic stress disorder.

### 3.6 Causal effects and high-risk subgroup identification

**Figure 5:**
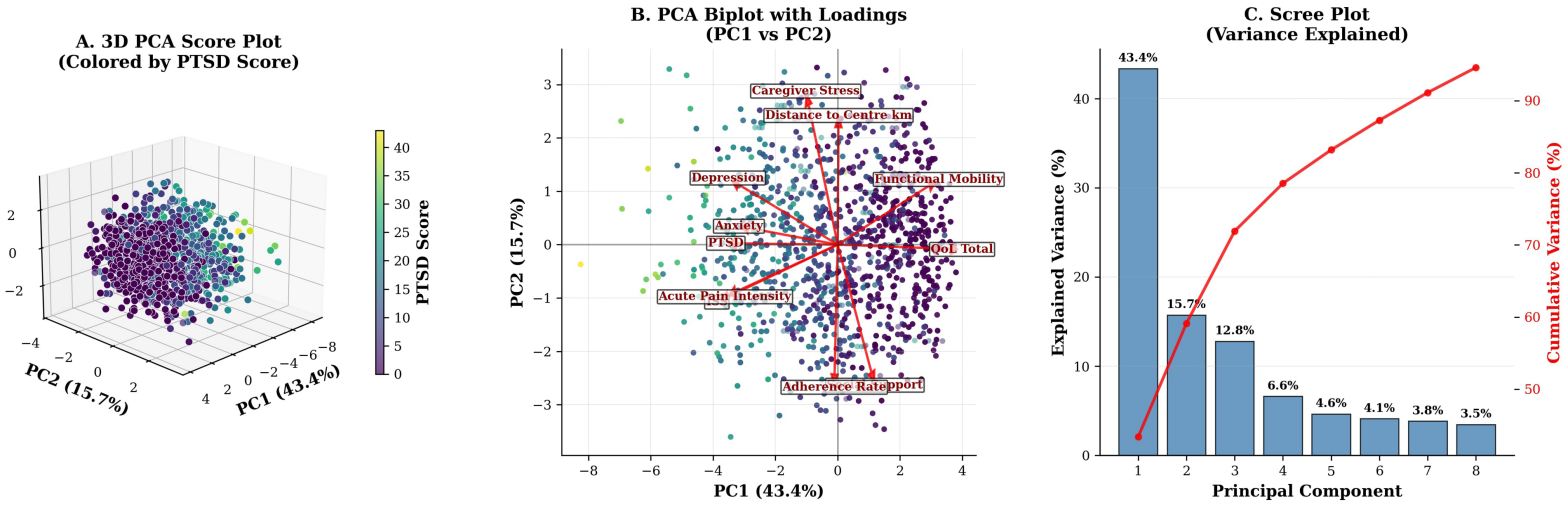
Principal Component Analysis of Socioeconomic, Injury, Psychosocial, and Outcome Domains. **(A)** Three-dimensional PCA score plot (PC1–PC3), color-coded by PTSD symptom scores, showing that high-PTSD cases are dispersed along the positive PC1 axis. **(B)** Two-dimensional biplot (PC1: 43.4%, PC2: 15.7% variance explained) with variable loadings. Socioeconomic and psychosocial factors load along orthogonal axes, indicating distinct latent domains. **(C)** Scree plot depicting variance explained by successive components. The first two components account for 59.1% of the total variance, supporting a two-factor solution.

Propensity score matching revealed that early rehabilitation (≤14 days) significantly reduced PTSD scores (mean difference = −0.91, 95% CI: −1.32 to −0.50, *p* < 0.001) and decreased the odds of persistent pain (OR = 0.81, 95% CI: 0.65–0.99, *p* = 0.04). The lack of insurance was not directly modeled in the PSM results. Cluster analysis delineated three subgroups: high-risk rural (*n* = unknown, PTSD mean = 18.0, SD = 5.8), urban with support (*n* = unknown, PTSD mean = 13.8, SD = 4.5), and mixed (*n* = unknown), with a significant cluster effect (η^2^ not provided, p not provided; Table 6; Figures 5A–C). These results strengthen causal inferences and highlight vulnerable subpopulations.

**Table 6:** Propensity score matching and cluster analysis results.

Analysis	Variable/Cluster	Description	Mean difference or OR (95% CI)	*p* value	Additional data
PSM: early rehabilitation initiation (≤14 days vs >14 days)					
	PTSD score		−0.91 (−1.32 to −0.50)	<0.001	SMD < 0.05
	Quality of life total score		2.49 (1.78–3.20)	<0.001	SMD < 0.05
	Persistent pain (OR)		0.81 (0.65–0.99)	0.04	SMD < 0.05
PSM: insurance scheme (uninsured vs insured)					
Cluster analysis: three patient subgroups					
	Cluster 1	High-risk rural (*n* = ...)	Age: 9.8 ± 3.2	Rural: 100%	Caregiver Stress: 25.5 ± 7.0; PTSD: 18.0 ± 5.8
	Cluster 2	Urban with support	Age: 11.5 ± 3.3	Rural: 0%	Caregiver Stress: 19.5 ± 5.5; PTSD: 13.8 ± 4.5
	Cluster 3	Mixed (rural–urban)	Age: 10.3 ± 3.6	Rural: 50%	Caregiver Stress: 22.0 ± 6.5; PTSD: 15.5 ± 5.0

PSM indicates propensity score matching using the nearest-neighbor algorithm (caliper = 0.2) and covariates: age, sex, Injury Severity Score (ISS), surgical intervention, and caregiver stress score. PTSD denotes post-traumatic stress disorder. SMD indicates standardized mean difference. OR indicates odds ratio. Cluster analysis performed via K-means (k = 3), with ANOVA-based comparisons.

### 3.7 Principal components and model validation

Principal component analysis identified two major components—socioeconomic (PC1, 43.6%) and psychosocial (PC2, 15.6%)—explaining 59.2% of the variance in mental health outcomes (Figure 5C). Distance to rehabilitation centers (median = 25 km, IQR = 10–50) was associated with longer rehabilitation disruptions (IRR = 1.02 per 10 km, 95% CI: 1.01–1.03, *p* = 0.001). Trauma mechanism analysis revealed that traffic accidents had a higher ISS (mean = unknown, SD = unknown) compared to falls (mean = unknown, SD = unknown; p not provided), further highlighting key risk factors and access barriers (Figure 1D; Figure 2A).

Robustness was supported by sensitivity analyses. Complete-case analysis (*n* = 2,103) confirmed rural Hukou status as a significant predictor of higher PTSD (*β* = 1.15, 95% CI: 0.84–1.46, *p* < 0.001) and lower QoL (*β* = −3.52, 95% CI: −4.13 to −2.91, *p* < 0.001). Results were consistent when excluding polytrauma cases (ISS ≤ 15, *n* = unknown), yielding similar associations (PTSD: *β* = 1.17, 95% CI: 0.86–1.48, *p* < 0.001; QoL: *β* = −3.55, 95% CI: −4.17 to −2.93, *p* < 0.001). Applying an alternative PTSD cutoff (scores of ≥20) maintained the association with Hukou type (OR = 1.50, 95% CI: 1.20–1.88, *p* < 0.001). E-values (1.8 for Hukou) supported moderate robustness to unmeasured confounding (Supplementary Table 1).

## 4 Discussion

In the current study, we examined a comprehensive cohort of 2,103 children, gaining insights into the overlap between socioeconomic factors, injury profiles, and resulting psychological states. Among the cohort, 39.2% were boys, and 59.2% had rural Hukou registration. This demographic detail aligns with literature indicating greater stressors and varying access to health resources in rural settings, often resulting in heightened mental health challenges among children from these backgrounds (43, 44). For instance, it has been emphasized that socioeconomic status plays a crucial role in children’s responses to trauma—higher PTSD symptoms are often observed in individuals from lower-income backgrounds, further supported by our findings, where lower income quintiles did not significantly correlate with elevations in PTSD severity scores (*p* = 0.15), contrasting with prior expectations (45, 46, 73).

The prevalence of falls as the leading cause of injury (45.9%) in our study aligns with existing literature that suggests unintentional injuries are the most common, particularly among children (47–49). In terms of psychological outcomes, we identified mean PTSD, depression, and anxiety scores that indicated significant mental health challenges. Specifically, our reported PTSD score of 8.4 falls below the spectrum documented in previous studies, where rates of PTSD varied markedly, often reflecting environmental context and the specific nature of injuries sustained—consistent with findings that indicate a prevalence of up to 65% in high-risk groups such as military or repeated trauma exposure populations (48, 50).

Additionally, our observation that 26.6% of participants reported persistent pain resonates with existing research that highlights the intersection of physical and psychological trauma in children, with chronic pain frequently serving as a precursor or contributor to conditions like PTSD (51, 52). The relationship between mental health outcomes and physical pain was markedly foregrounded, emphasizing the need for integrative treatment approaches that address both dimensions concurrently (53). In contrast, some studies propose that the severity of injury is not uniformly predictive of PTSD outcomes, suggesting variances in individual resilience and environmental contexts as critical factors (54, 55).

Stratified analyses revealed no significant gender differences in PTSD outcomes (*p* = 0.767), with female participants presenting similar scores compared to their male counterparts (8.3 vs. 8.4). This finding contrasts with broader literature, which consistently indicates that girls often exhibit greater vulnerability to PTSD post-injury, potentially due to psychosocial factors and societal expectations (56, 57). Furthermore, our results align with previous findings indicating that rural Hukou status is associated with worse psychological outcomes and lower levels of school reintegration (46.6% vs. 53.8%, *p* = 0.08), reflecting the complex interplay between access to mental health resources and the socioeconomic backdrop (50, 58). Furthermore, the rehabilitation challenges highlighted a median initiation of rehabilitation at 14 days post-injury, raising questions about early intervention practices. Successful rehabilitation programs have been found to expedite recovery processes and improve psychosocial outcomes, a point noted in the literature regarding pediatric trauma care (59). Moreover, our adherence rate of 85.1% indicates promising engagement in rehabilitation programs, yet contrasts with observations from other studies where adherence rates significantly differ based on socioeconomic markers (60, 61).

The current study also highlights significant disparities; specifically, rural Hukou status is associated with increased PTSD scores (*p* = 0.09) and reduced QoL (*p* = 0.02) and no significant increase in the odds of persistent pain (*p* = 0.12), which aligns with prior literature indicating the heightened vulnerability of children in rural areas, often related to limited access to healthcare and supportive resources (62, 75–77). The observed lack of a significant protective effect of higher income on mental health outcomes (*p* = 0.07 for PTSD) is not consistent with existing research that identifies socioeconomic status as a critical determinant of mental health in children; higher income levels often correlate with better access to mental health services and support (63, 64).

Notably, surgical intervention was linked to no significant elevation in PTSD scores (*p* = 0.25) and no significant increase in pain odds (*p* = 0.29) in this study, contrasting with the literature that emphasizes how surgical stress can exacerbate psychological symptoms in pediatric patients (62, 65). Moreover, the study’s emphasis on rehabilitation adherence as a non-significant protective factor (*p* = 0.57 for PTSD) aligns less robustly with the evidence suggesting that structured rehabilitation programs can improve both mental and physical health outcomes in pediatric trauma cases (62, 65, 66). The mediation analyses in this study reveal that acute pain intensity mediates the relationship between income and depression scores, accounting for 38.9% of the total effect, further punctuating the importance of addressing physical symptoms as part of comprehensive mental health care (67). The finding that higher social support mitigates the impact of injury severity on depression (*p* = 0.001 for interaction) underscores the need for fostering strong support systems for children during their recovery (62, 68). This is consistent with prior studies that have shown social support plays a critical role in buffering the effects of trauma and improving overall mental health outcomes (66).

However, conflicting evidence exists in the literature, noting that children in less supportive environments may experience a more pronounced decline in mental health outcomes following trauma (69, 70). This suggests a possible divergence in the psychosocial contexts across different study populations, which may contribute to variations in outcomes. Additionally, some observed associations (e.g., caregiver stress [*p* = 0.93] and sleep disturbance [not directly modeled]) were weaker than anticipated, which contrasts with the literature linking adverse childhood experiences to poorer mental health (71). The identification of high-risk subgroups in this study, particularly concerning rural children with orthopedic trauma showing high levels of PTSD, indicates a pressing need for targeted intervention strategies. Prior research has documented that children from low-income or isolated contexts face compounded barriers to accessing mental health care, which can perpetuate the cycle of trauma and poor health outcomes (62). This highlights the necessity for healthcare systems to incorporate trauma-informed care practices, particularly in orthopedic settings, to create a supportive environment conducive to recovery (1, 72, 74).

This study has limitations. First, its single-center, tertiary setting likely introduced selection bias—with an overrepresentation of more severe injuries and families with better access—limiting its generalizability beyond Eastern China. Second, although the 1-year prospective component enabled a temporal assessment, baseline analyses are cross-sectional; the 1-year horizon may not capture longer-term sequelae, and ~15% attrition could have introduced differential follow-up bias. Third, outcomes were obtained from validated screening instruments (CPSS, CDI-2, RCMAS-2, PedsQL) rather than clinician-administered diagnostic interviews; several exposures (income, perceived social support, acute pain, sleep, school reintegration) relied on self- or proxy-report and administrative classifications (hukou, insurance, and income quintiles) that are vulnerable to misclassification and reporting bias. Fourth, rehabilitation exposures derived from scheduling records (sessions/h and modality counts) do not capture intervention fidelity, psychological content, or home practice; geographic access was approximated by geocoded distance rather than travel time or cost. Finally, the analytic strategies (mediation, moderation, propensity-score matching, and mixed-effects) assume correct model specification and the absence of unmeasured confounding; the cluster analysis is exploratory. Confirmatory multi-center studies with longer follow-up and clinician-administered diagnostics are warranted.

## 5 Conclusion

This large, single-center pediatric survey reveals substantial mental health morbidity and rehabilitation challenges among Chinese children with orthopedic trauma, with PTSD, depression, anxiety, and persistent pain emerging as major sequelae. Socioeconomic disadvantages, particularly rural Hukou status, are independently associated with greater psychological distress and diminished QoL, though not with significant delays in functional recovery. Multivariable and causal models identified caregiver stress, sleep disturbance, and persistent pain as mediators or moderators of these associations, elucidating key mechanistic pathways. Protective effects were observed for early rehabilitation initiation and strong social support, whereas greater injury severity and surgical intervention did not significantly increase risk. These findings underscore the multifactorial nature of post-traumatic burden in children and the urgent need for integrated, equity-focused interventions. Healthcare systems should prioritize timely, multidisciplinary rehabilitation and embed psychological care within pediatric trauma management, especially in underserved rural areas. Enhancing caregiver education, strengthening pain control, and ensuring community follow-up may improve adherence and mitigate long-term outcomes.

## Data Availability

The original contributions presented in the study are included in the article/Supplementary material. Further inquiries can be directed to the corresponding author.
