# Supplementary material for: Mental health outcomes and rehabilitation challenges in children with orthopedic trauma: a public health survey from a pediatric rehabilitation center
Source: Front Public Health. 2025 Oct 22;13:1652569. doi: 10.3389/fpubh.2025.1652569 (PMC12586071; doi:10.3389/fpubh.2025.1652569)
Supplement: Supplementary file 1 [file Table_1.DOCX]

**eTable 1. Sensitivity Analyses for Primary Outcomes.**

| **Analysis** | **Predictor** | **PTSD Score** | **Quality of Life Total Score** |
| --- | --- | --- | --- |
|  |  | β (95% CI) | β (95% CI) |
|  |  | P Value | P Value |
| **Complete-case analysis (n = 3465)** |  |  |  |
|  | Hukou type (rural vs urban) | 1.18 (0.82 to 1.54) | −3.45 (−4.05 to −2.85) |
|  |  | <.001 | <.001 |
|  | Income quintile | −0.43 (−0.58 to −0.28) | 1.75 (1.45 to 2.05) |
|  |  | <.001 | <.001 |
| **Excluding polytrauma (ISS ≤15, n = 3100)** |  |  |  |
|  | Hukou type (rural vs urban) | 1.15 (0.78 to 1.52) | −3.40 (−4.00 to −2.80) |
|  |  | <.001 | <.001 |
|  | Income quintile | −0.40 (−0.55 to −0.25) | 1.70 (1.40 to 2.00) |
|  |  | <.001 | <.001 |
| **Alternative cutoff (PTSD score ≥20)** |  | OR (95% CI) |  |
|  |  | P Value |  |
|  | Hukou type (rural vs urban) | 1.50 (1.20 to 1.88) | — |
|  |  | <.001 |  |
|  | Income quintile | 0.82 (0.75 to 0.90) | — |
|  |  | <.001 |  |
| β indicates regression coefficient; OR, odds ratio; ISS, Injury Severity Score; PTSD, posttraumatic stress disorder. Models adjusted for all predictors listed in Table 4. E-values for unmeasured confounding: 1.8 (hukou type), 1.6 (income quintile). Benjamini-Hochberg adjusted P values reported for secondary outcomes. | | | |
